# 

**Published:** 1889-11-30

**Authors:** 


### NOTICE TO CORRESPONDENTS.

All MS., Letters, Books for Review, and other matters intended for the Editor, should be addressed The Editor, The Lodge, Porchester Square, London, W.

### Notice to Advertisers and Subscribers.

All Advertisements, Orders for Copies of The Hospital, and Business Communications should be addressed to The Publisher, *not to the Editor*, The Hospital, 139-140, Salisbury-court, Fleet-street, E.C.

### To Residents, Secretaries, and Matrons

The Editor will be glad to have the Regulations and each Report as issued by Asylums, Hospitals, Convalescent and Nursing Institutions, as well as those of other Charities, and an early copy in duplicate of all Appeals and Festival Notices, etc., addressed to *The Editor, The Lodge, Porchester-square, London, W.* Any superintendent, secretary, matron, or other chief official who regularly sends such information may be registered, on application to the Publisher, to receive free of cost a cover for binding The Hospital in half-yearly volumes.

### The Students' Column.

### UNIVERSITY OF LONDON.

#### M.B. EXAMINATION, OCTOBER, 1889.

Pass List.—*First Division.*—Henry Moore Bowman, St. Bartholomew's; Rubert William Boyce, University College; Hubert Carpenter Bristowe, St. Thomas's; Henry Johnstone Campbell, Guy's; Percy James Duncan, Charing Cross; Frank Fawssett, St. Thomas's; William Soltau Fenwick, London Hospital and Strasburg and Berlin; John Arthur Hayward, St. Bartholomew's; Charles Arthur Kent, *B.Sc.*, University College; Charles MacGowan Kitching, Guy's; Joseph Johnston Macgregor, St. Bartholomew's; Robert Devereux Mothersole, Guy's; John Edward Platt, Owens College and Manchester Royal Infirmary; Charles Frederick Seville, Owens College and Manchester Royal Infirmary; Edgar Olive Turner, University College.

*Second Division.*—Horatio George Adamson, St. Bartholomew's; Launcelot William Andrews, St. Bartholomew's; George Henry Barker, *B.Sc.*, Bristol Medical School; George Black, Guy's; Richard Oxley Bowman, Owens College and Manchester Royal Infirmary; Walter Henry Brazil, Owens College; James Joseph Buist, St. Bartholomew's; Frank Calder, Bristol Medical School; Frances May Dickinson, London School of Medicine for Women; Charles Duer, University College; Thomas James Dyall, St. Bartholomew's; Alfred Downing Fripp, Guy's; Thomas Ashton Goodfellow, Owens College and Manchester Royal Infirmary; Albert Green, Guy's; William Griffiths, *B.Sc.*, Middlesex; Julius Hamel, University College; Alan Jasper Heath, University College; Philip Henry Hensley, King's College; Ernest Wickham Hore, University College; Charles Rowe Killick, London; Henry Ernest Knight, St. Bartholomew's; Mary A. McCulloch Knight, London School of Medicine for Women; Milton Prentice Ledward, Owens College and Manchester Royal Infirmary; Charles Pardey Lukis, St. Bartholomew's; George John Padbury, Guy's; Edward Percy Paton, St. Bartholomew's; Ransom Pickard, St. Bartholomew's; George Henkell D. Robinson, St. Bartholomew's; Joseph Crofts Rossall, St. Mary's; Arnold Scott, Guy's; John Alexander Shaw-Mackenzie, University College; Ernest Hugh Snell, *B.Sc.*, Queen's College, Birmingham; Frank Barber Wells, University College; John Price Williams, Owens College and Manchester Royal Infirmary; Robert Edwin Williams, Guy's.

### Scraps and Gleanings.

Kidderminster Infirmary has invested £1,000.

At Dewsbury a man, aged 73, has died of hydrophobia.

Burton Hospital Saturday collection amounted to £673.

Hot-water operation-tables are to be the fashion in future.

Fourteen infants died last week in London from "overlaying."

A mission to medical students began last Monday at Bishopsgate, and concludes on the 30th.

Huddersfield is still much exercised about the proper site for the proposed fever hospital.

The Ladies Committee of Mexborough Montagu Cottage Hospital have collected £50 by a tea.

The death is announced of Miss Fanny Jane Butler, medical missionary at Srinigar, Kashmir.

The Lock Hospital, Harrow-road, issues a special appeal for £1,000 to free the institution from debt.

Dublin Hospital for Incurables had 23 applicants for five vacancies at their last monthly meeting.

Last quarter the death rate in England and Wales among men was 17·7; among women it was only 15·7.

The Skinners' Company has granted a donation of £10 10s. to the Deaconesses' Institution and Hospital, Tottenham.

The Mayor of Liverpool attended the anniversary service in aid of the Southern Hospital. The collection amounted to £134.

There are nearly 40 places of worship in Liverpool where no collection was made for the Hospital Sunday Fund. This ought not to be.

Dr. Murray Lindsay has written to the *Derby Mercury* explaining his objection to the infirmary rules which disqualify Irish and Scotch M.D.'s.

Princess Christian is being treated at Wiesbaden by Dr. Pagenstecher, the oculist who was so successful with Sir Michael Hicks-Beach.

The question of an out-patient system is to the fore at Newcastle Royal Infirmary. We hope it will be carried in February at the annual meeting.

An entertainment was held at Seaforth on the 18th inst. in aid of the local dispensary. There was a good attendance, and the amateur minstrels sang well.

The centenary of John Howard, the great prison reformer, will be celebrated on January 20th. Dr. J. Coombs, the Bishop of London, and others are aiding the movement.

Serious complaints are being made in the City with regard to the Stock Exchange building. Three or four members, it is said, have recently died from typhoid fever.

Cholera is increasing in Kermanshah, and is still prevalent in Khorremabad and Hamadan and the intermediate districts. The epidemic is spreading in an easterly direction.

Mr. H. K. Lewis announces the publication on December 2nd of an important work in two volumes by Professor Edgar Crookshank, entitled the "History and Pathology of Vaccination."

Dr. Simon, the public medical officer of health for Singapore, is agitating for the establishment of a medical school for the Straits Settlement in that town, and hopes for Government support.

The authorities of Schleswig-Holstein have exempted from taxation all the dogs which, "sleeping in the beds of their masters or mistresses, serve to prevent them from gout, rheumatism, and other ailments."

Hairdressers are opposed to the Act being brought forward by chemists to prevent poisons being sold by any save members of the Pharmaceutical Society. Hair-dyes and washes often contain poison.

Last Saturday Lady Clark cut the first sod of the International Exhibition of Electrical Engineering and General Industries which is to be held in Edinburgh next year, in commemoration of the opening of the Forth Bridge.

At the last meeting of the Royal Commission on Vaccination, Dr. Thorne Thorne, assistant medical officer to the Local Government Board, was examined at considerable length as to the administration of the Vaccination Acts.

Edward Donohue, a labourer employed at the chemical works of Messrs. Bramwell and Sons, St. Helens, died at the Providence Hospital in that town on Saturday, from injuries received by falling into a tank of boiling Epsom salts.

Some of the worst quarters of New York City have been lighted with electric light, and a great decrease in crime has resulted. An American lady now visiting London proposes that our dark alleys and archways should be similarly lighted up.

A lecture on the life and career of General Charles Gordon was delivered at the Town Hall, Kensington, on Tuesday. The proceeds of the lecture will be divided between the Gordon Boys' Home, Chobham, and Gordon Hospital at Cyprus.

Two accidents at football have taken place at Belfast. In the first place, a young man named John M'Bain had his leg broken while playing against the Black Watch; and in the second, a player named Calvert sustained a fracture of the kneecap.

The case of a very large family came before the Freemasons' Board of Benevolence lately, when the sum of £805 was voted in grants to petitioners in distress. One petitioner, who was 67 years of age, has had 39 children, the youngest of this interesting family being only three months old.

The sale of Mr. Rennell Rodd's book, "Frederick, Crown Prince and Emperor," to which an introduction was written by the Empress Frederick, realised a profit of £400. This sum the Empress has, in accordance with her previously expressed intention, sent as a donation to the Hospital for Diseases of the Throat in Golden-square.

The Examination for the Certificate in Psychological Medicine will take place at Bethlem Hospital, London, on the 19th and 20th of December; at the Royal Asylum, Morningside, Edinburgh, on the 16th of December; at the Royal Asylum, Aberdeen, on the 20th of December; and at the Richmond Asylum, Dublin, on the 4th of December.

